# Leaf, root, and soil microbiomes of an invasive plant, *Ardisia crenata*, differ between its native and exotic ranges

**DOI:** 10.3389/fmicb.2023.1302167

**Published:** 2023-11-22

**Authors:** Naoto Nakamura, Hirokazu Toju, Kaoru Kitajima

**Affiliations:** ^1^Graduate School of Agriculture, Kyoto University, Kyoto, Japan; ^2^Center for Ecological Research, Kyoto University, Otsu, Japan

**Keywords:** bacteria, fungi, exotic invasive species, leaf endophytes, functional guilds, next generation sequencing, plant-microbe interactions, soil microbial community

## Abstract

**Introduction:**

Ecological underpinnings of the invasion success of exotic plants may be found in their interactions with microbes, either through the enemy release hypothesis and the enhanced mutualism hypothesis. Whereas recent high-throughput sequencing techniques have significantly expanded our understanding of plant-associated microbiomes and their functional guilds, few studies to date have used these techniques to compare the microbiome associated with invasive plants between their native and exotic ranges.

**Methods:**

We extracted fungal and bacterial DNA within leaf endosphere, root endosphere and soil of an invasive plant, *Ardisia crenata*, sampled from their native range Japan and exotic range Florida, USA. Using Illumina sequencing data, we compared microbial community compositions and diversity between the native and exotic ranges, and tested whether abundance of pathogenic or mutualistic microbes differ between the native or exotic ranges in accordance to the enemy release hypothesis or the enhanced mutualism hypothesis.

**Results:**

Fungal and bacterial community compositions differed among leaves, roots and soil, and between the native and exotic ranges. Despite a higher microbial diversity in the soil in the exotic range than in the native range, the microbial diversity within leaf and root was lower in the exotic range compared to the native range. In addition, leaves in the native range harbored a greater number of plant pathogenic fungi compared to those in the exotic range.

**Discussion:**

These patterns suggest plant controls over what microbes become associated with leaves and roots. The higher abundance of leaf pathogenic fungi, including the pathogen which is known to cause specific disease in *A. crenata* in the exotic range than in the native range, support the enemy release hypothesis and highlighted potential importance of examining microbial communities both above- and below-ground.

## Introduction

Plant-microbe interactions are increasingly recognized to play a role for the reason why some exotic plants become invasive (Dawson and Schrama, 2016; Dickie et al., 2017; Egidi and Franks, 2018; Pearson et al., 2018). Since Elton (1958) published the concept of the “enemy release hypothesis,” many consider that the presence/absence of host-specific natural enemies is key to explain the increased invasiveness of organisms (Keane and Crawley, 2002; Catford et al., 2009; Jeschke et al., 2012; Heger and Jeschke, 2014). According to the enemy release hypothesis applied to plant-microbe interactions, a plant population may be kept at check by host-specific natural enemies in its original range, but the lack of these microbes in the exotic range endows a competitive advantage to non-native plant species (Adams et al., 2009; Chiuffo et al., 2015; Aldorfová et al., 2020; Huang et al., 2020; Kuźniar et al., 2020). Not only pathogenic microbes, but also differences in the geographical distribution of mutualistic microbes may explain invasion success by plants, for example, via novel associations with mutualistic microbes that enhance plant defense, growth and stress tolerance (“enhanced mutualism hypothesis”; Callaway and Ridenour, 2004; Traveset and Richardson, 2014; Aslani et al., 2019).

Despite the relevance of the enemy release and enhanced mutualism hypotheses in plant-microbe interactions between native and exotic ranges, only a few studies have compared the biogeography of microbial communities associated with invasive species (Harrison and Griffin, 2020). In such comparative studies, two aspects need to be considered. Firstly, in a given region, a plant species interact with many different microbes that interact with each other. Secondly, what microbes become associated with a given plant species in a given place depends not only on the microbial community composition in the environment, but also on specific parts where microbes may harbor (e.g., leaves, roots, and soil). If done properly, a comparison of how a given invasive plant species interacts with microbes between its native and exotic ranges in leaves, roots, and soil should shed light on the basic ecology of plant-microbial interactions, beyond the search for the key ecological process that explains its invasiveness.

To date, many studies on interactions between invasive plants and microbes have focused on soil microbiomes (Chiuffo et al., 2015; Aldorfová et al., 2020). Certain mutualistic microbes in the roots and rhizosphere are known to enhance stress tolerance to salt, heat, or resistance to plant pathogens (Rodriguez et al., 2009; Jogaiah et al., 2013), subsequently leading to successful plants invasion (Kamutando et al., 2017). Soil microbial communities vary geographically (Castellanos et al., 2009), and so do root endophytes (Brigham et al., 2023). Yet, few have investigated geographical differences in root-associated microbial communities of invasive plant species between its native vs. exotic ranges. Plants can recruit beneficial microbes to the rhizosphere with root exudates (Upadhyay et al., 2022), and control which microbes in the rhizosphere can penetrate the roots through immune responses and/or biofilm formation (Vieira et al., 2020). It is postulated that such selectiveness exerted by plants involves species-specific genetic factors (Bulgarelli et al., 2013; Edwards et al., 2015; Yu and Hochholdinger, 2018). Hence, if an exotic plant species has a strong coevolved relationship with certain soil microbes in its native range, a reduction in root-associated microbial diversity might be observed in the exotic range due to a scarcity of microbes that can enter roots. Additionally, a local monodominance of an invasive plant could lead to a decrease of local plant diversity, resulting in a reduction of microbial species richness in the rhizosphere (Lu-Irving et al., 2019).

Similarly to root-associated microbes, certain leaf endophytes are shown to benefit the host plant performance by protecting against pathogens and increasing resistance to insect herbivory (Arnold et al., 2003; Tanaka et al., 2005). Yet, leaf endophytes receive much less attention than soil microbes do in invasive plant research. Hence, understanding how differences in microbial composition and diversity between native and exotic ranges influence plant invasiveness remains largely unexplored (but see Lu-Irving et al., 2019; Pan et al., 2023). Experimental evidence from field and manipulative studies suggest that the leaf microbial composition is influenced more by the surrounding environmental microbial pool than by plant genetic factors (Whitaker et al., 2018; Pan et al., 2023). Therefore, deciphering how leaf microbial communities associated with leaves and roots of an invasive plant species differ between its native and exotic ranges will help fill a research gap relevant for both the enemy release hypothesis and the enhanced mutualism hypothesis.

In recent years, amplicon sequencing technologies allow high-throughput analyses of microbial taxa/species belonging to diverse functional guilds such as pathogens and mutualists (Nguyen et al., 2016). This approach has certain advantages over the more traditional approach of comparing differences in plant growth between sterilized and non-sterilized soils (Dawson and Schrama, 2016). Whereas sterilization experiments can provide useful hints as to the importance of microbial communities in the soil and leaf litter (which is the major spore source of leaf endophytic fungi), the effect detected is a net effect of negative and positive effects from pathogens and mutualists (e.g., Pizano et al., 2019). Comparisons of microbial functional guild compositions between their native and exotic ranges can be informative as to how the abundance of pathogens and mutualists differ between the geographic ranges.

In this study, we chose *Ardisia crenata*, a shade tolerant shrub native to East Asia that acts as an aggressive invader in North America (Dozier, 2000; Kitajima et al., 2006). It can form a mutualistic association with arbuscular mycorrhizal (AM) fungi found in the mesic forests that it invades in North Central Florida, USA (Bray et al., 2003). Although *A. crenata* occurs at low densities in its native range in Japan (no more than a few adults within 5 m of each other), it forms a dense monodominant understory in Florida (Supplementary Table S1), reducing the diversity of native plant species. The lack of noticeable herbivores or seed predators in both ranges (Kitajima et al., 2006) suggests that differences in plant-microbe interactions between native and exotic populations may be an important factor underpinning its invasion success. *A. crenata* is widely cultivated in Japan, but cultivation at high density often results in an onset of heavy mortality within Japan. These pieces of background information make *A. crenata* to be an ideal candidate for studying microbiome differences between the native range (Japan) and exotic range (Florida). We described and compared the diversity and structure of fungal and bacterial communities in leaves, roots, and soil with high-throughput Illumina sequencing. Furthermore, we assigned microbes to functional guilds (pathogens, mutualists) and compared taxonomic composition within each microbial functional guild. We hypothesized that (1) microbial community structure would differ by plant parts and geographical ranges, (2) microbial α diversity would be lower in the exotic range than in the native range, because of the monodominance of *A. crenata* in the former, (3) the observed differences in functional taxa between native and exotic ranges would align with either the enemy release hypothesis or the enhanced mutualism hypothesis. The results would be corroborative of the enemy release hypothesis if putative pathogens are more prominent in the native range than in the exotic range. Conversely, the enhanced mutualism hypothesis is supported if we detect positive patterns supportive of the possibility that *A. crenata* in the exotic range has formed novel associations with beneficial microbes.

## Materials and methods

### Study sites and sampling

**Figure 1:**
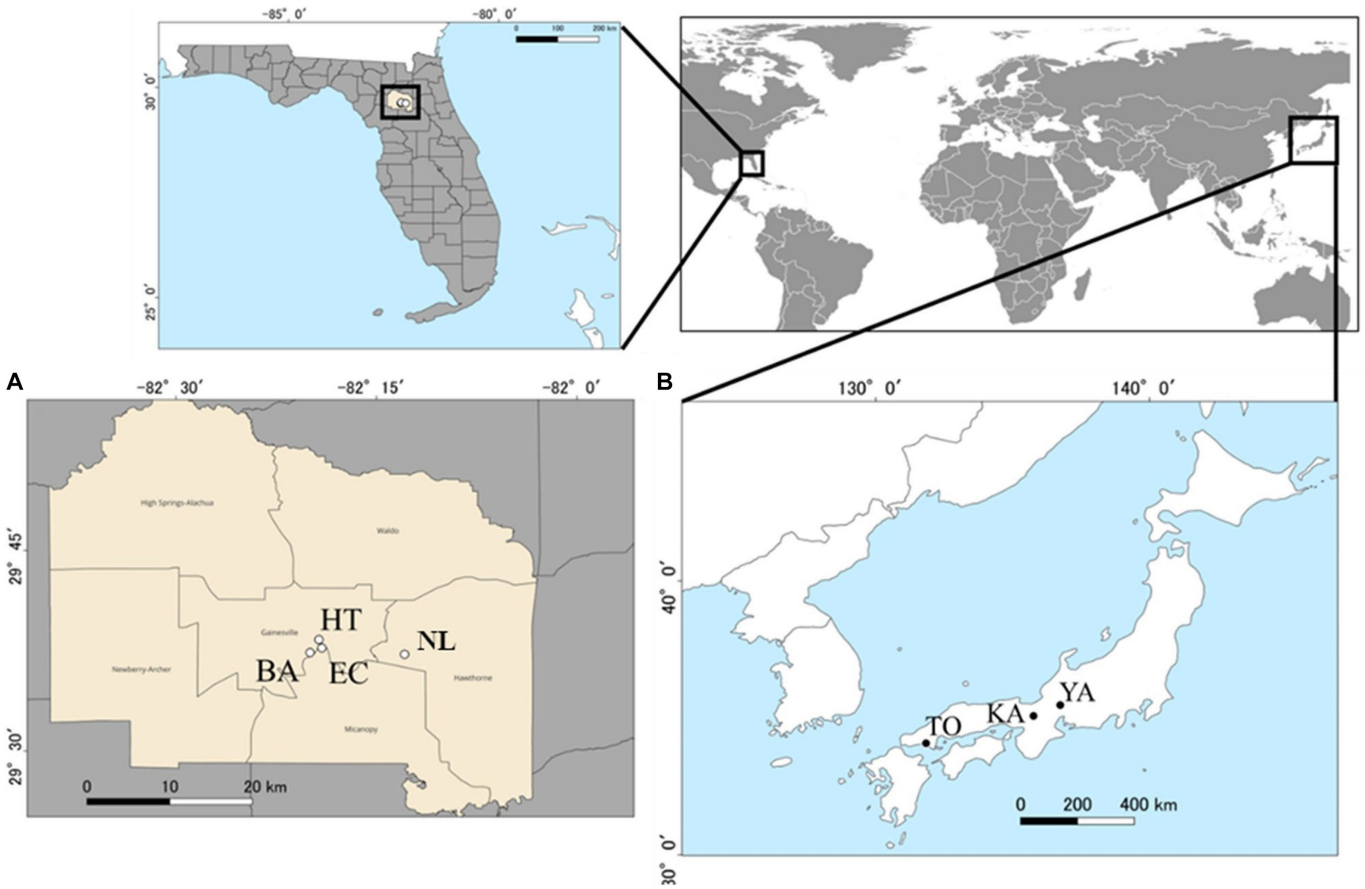
Sampling sites (circles) for this study within the exotic range (4 locations in Alachua County, Florida) [**(A)** open circles], and the native range (three locations in Japan) [**(B)** closed circles]. See Supplementary Table S1 for the full names of locations abbreviated in two letter codes. County layers map (TIGER/Line Shapefile, 2016, state, Florida, Current County Subdivision State-based) and city locations (TIGER/Line Shapefile, Current, State, Florida, Places) were accessed from the United States Census Bureau, https://catalog.data.gov/dataset.

We set three and four sampling sites each in the native range (Honshu, Japan) and exotic range (Florida, United States), respectively (Figure 1). Sampling sites in the native range were Kamigamo Experimental Station (KA) of Kyoto University, Tokuyama Experimental Station (TO) of Kyoto University, and Yanagido Experimental Station (YA) of Gifu University, all of which have a warm temperate climate (mean annual temperature of 15.7–17.7°C), mean annual precipitation of 1522.9–2625.5 mm (from observation from1991 to 2020, Japanese meteorological agency). All four sites in the exotic range were in Alachua County, Florida (mean annual temperature of 20.7°C, mean annual precipitation of 1227.1 mm) (1991–2020, Florida Climate Center). They were Bivens Arm Nature Park (BA), Evergreen Cemetery (EC), Hawthorne Trail (HT), and Newnan’s Lake (NL). See Supplementary Table S1 for geographical coordinates and vegetation characteristics of these sites. Kitajima et al. (2006) has reported genetic differences between the invasive population in Florida and wild populations of *A. crenata* in Kyushu and Okinawa. In contrast, the wild populations of *A. crenata* in Honshu sampled in the current study are genetically close to the invading populations in Florida both in terms of morphological traits and DNA sequences analyzed with ddRAD-seq (Wataru Noyori, unpublished data). Hence, differences in microbial communities associated with *A. crenata* individuals in the current study are unlikely to reflect differences in plant genotype, but likely due to geographical differences in the background microbial communities and/or differences in local density of *A. crenata* individuals (low vs. high density in Japan vs. Florida).

At each sampling site, two transects, 1 m wide and 15 m long each, were set to encompass the highest local density of *A. crenata*. The two transects were separated by a minimum of 10 m from each other. Each transect was divided into 1 m × 1 m quadrats, from which we collected a healthy individual of *A. crenata* with height less than 15 cm (a total of 30 per site). From each plant sampled, we collected three leaves without disease, five fine roots as well as *ca.*10 g soil from its vicinity (within 5 cm). The total number of plants sampled was 210 (30 individuals × 7 sites). In the field, samples were individually sealed in plastic bags, and kept immediately in a cooler box with ice packs until further processing in the laboratory. In the lab, leaf and root samples were surface sterilized for 1 min in water with an ultrasonic cleaner, followed by sequentially soaking in 70% ethanol for 1 min, 0.5% NaClO for 1 min, and sterile water for 1 min. Afterward, all samples were sealed in plastic bags with silica gel to dry and store at the −20°C freezer until DNA extraction.

### DNA extraction and sequencing

From each leaf sample, we cut a 1-cm^2^ piece to include leaf edge with a pair of sterile scissors. For root samples, three 2 cm long pieces of fine root were cut with a pair of sterile scissors. These samples were pulverized with Qiagen Tissue Lyser II (at 25/s, for 2 min, Qiagen) with two 4 mm zirconium beads inside 1 mL lysis buffer (20 mmol/L Tris, pH 8.0, 2.5 mmol/L EDTA, 0.4 mol/L NaCl, 0.05% SDS). For extraction of DNA from soil, 0.25 mL of each sample was placed in a 2 mL microcentrifuge tube containing 900 μL lysis buffer and 100 μL skim milk, and 0.25 mL of 0.5 mm zirconium beads, then pulverized with Qiagen Tissue Lyser II (at 25/s, for 2 min). After centrifugation at 4,400 rpm for 5 min, we collected the supernatant containing extracted DNA for PCR intended for amplifying the 16S region for procaryotes. Because this supernatant could not yield successful PCR for fungal ITS, we also used the phenol-chloroform extraction method (Wilson, 2001) for PCR intended for the ITS region of fungi.

The prokaryotic 16S rRNA and fungal internal transcribed space 1 (ITS1) regions were PCR-amplified following the protocol detailed elsewhere (Toju et al., 2019) with some modifications. Briefly, for the prokaryotic 16S rRNA region, the primer set 515f/806rB (515f, Caporaso et al., 2011; 806rB, Apprill et al., 2015) was fused with the Illumina sequencing primer region and 3-6-mer Ns for improving sequencing quality (Lundberg et al., 2013). Likewise, the fungal ITS1 region was amplified using the primer set ITS1-F_KYO1/ITS2_KYO2 (Toju et al., 2012) fused with the Illumina sequencing primer region and 3-6-mer Ns. We conducted PCR with the DNA polymerase system of Ampdirect Plus (Shimadzu, Kyoto, Japan) with a temperature profile of 35 cycles consisting of denaturation at 98°C for 10 s, annealing at 55°C for 60 s, extension at 72°C for 60 s, and a final extension at 72°C for 7 min, for both primers. Fusion primers with P5/P7 Illumina adapters and 8-mer index sequences for sample identification were added to the PCR products. In the reaction, the DNA polymerase system of KOD One (Toyobo) was used with a temperature profile of 8 cycles at 98°C for 10 s, 55°C for 5 s, 68°C for 30 s, and a final extension at 68°C for 2 min. The amplified PCR fragments were purified and equalized using AMpure XP Kit (Beckman Coulter), and equal volumes of all specimens were pooled together. The pooled library was sequenced with the Illumina MiSeq sequencer with 10% PhiX spike-in (Center for Ecological Research, Kyoto, Japan, 2 × 300 cycles).

### Bioinformatics

The bcl2fastq 1.8.4 program distributed by Illumina was used to convert the raw sequence data into FASTAQ files. The FASTAQ files were demultiplexed with the program Claident v0.2.2018.05.29 (Tanabe and Toju, 2013). Chimeric and low quality-score reads (< 20) were subsequently discarded and the reads that passed the filtering process were clustered using VSEARCH (Rognes et al., 2016) with 97% clustering threshold as implemented in Claident. Then, operational taxonomic units (OTUs) were obtained, resulting in a total read number of 4,040,099 and 3,556,515 from the 16S and ITS1 primers, respectively. Taxonomic assignment of OTUs was performed with a combination of the query-centric auto-*k*-nearest neighbor (QCauto) method (Tanabe and Toju, 2013) and the lowest common ancestor (LCA) algorithm (Huson et al., 2007) as implemented in Claident. Afterward, unclassified bacterial and fungal OTUs at the kingdom level were subjected to a blastn search, and OTUs matching of plant-derived sequences (chloroplast-derived 16S) were removed. Note that the 16S 515f/806rB primers used in this study also amplified host-derived sequences from leaf and root samples (around 60% of all 16S reads). For 16S reads of leaf samples, about 90% of the post-filter reads clustered into closely related OTUs affiliated with *Burkhorderia crenata*, a known symbiont in the leaf-edge nodules of *A. crenata* (Carlier et al., 2016). Many of these OTUs could be identified with nonuniform descriptions like “symbiont bacteria” in the public database. Thus, we attempted a phylogenetic approach to conclude that these OTUs as “*Burkholderia* sp.” for use in subsequent analysis after confirming the blastn results of those OTUs were within monophyletic group of *B. crenata* (Supplementary Figure S1). Finally, we excluded all OTUs that could not be assigned to either bacteria or fungi at the kingdom level (i.e., archaea or unidentified taxa), and then proceeded with the following statistical analyses.

To minimize the effect of PCR/sequencing errors, we removed the OTUs that represented less than 0.1% of the total reads in each sample (Peay et al., 2015). This resulted in high quality reads of 982,788 and 3,264,380 for 16S and ITS primers, respectively. The dataset was rarefied at 200 reads and 1,000 reads per sample for bacteria and fungi, respectively, with the “rrarefy” function of the “vegan” package (Oksanen et al., 2022) of R version 4.2.1 (R Core Team, 2022). Samples that yielded less than these read numbers were discarded, leaving bacterial 499 and fungal 469 samples. See Supplementary Table S2 for the sample size for each site. The rarefaction curves for each group reached to an asymptote (except for 16S in the soil), indicating that we had adequate levels of sampling (Supplementary Figure S2). After rarefaction, 4,819 and 3,998 unique OTUs of bacteria and fungi were included in the final analysis. Of these, 4,021 (83.4%) and 3,933 (98.4%) could be classified at the phylum level, and 1,603 (33.3%) and 1,653 (41.3%) were classified at the genus level for 16S and ITS reads, respectively. Further blastn analysis based on the NCBI database was performed on several OTUs to identify them at the species level. Fungal OTUs were assigned their ecological functional guilds including pathogens and mutualists (i.e., plant pathogen, AM fungi) based on their taxonomic assignment, with the FUNGuild (Nguyen et al., 2016). Only OTUs with a confidence rank of “High Probability” or “Probability” were retained in the analysis, but those with “Possible” and those assigned to more than one guild were treated as “Unidentified.” This resulted in 1,213 of 2,785 OTUs assigned guilds (30.5%), with remaining 69.5% being unidentified. Finally, for fungal and bacterial OTUs that account for more than 2% of the total relative abundance at the genus level, a literature survey based on the published literature was conducted on functional taxonomic guilds.

### Statistical analysis

We calculated Shannon diversity of fungal and bacterial communities was calculated as the exponent of Shannon entropy (i.e., $\exp\left(-\sum P_{i}\;\ln\left(P_{i}\right)\right)$ ; where *Pi* is the proportional abundance of species *i*, Shannon, 1948) calculated from the R package “vegan.” To test differences among plant parts and between the native and exotic ranges, ANOVA was conducted and a *post hoc* comparison with a nonparametric Kruskal-Wallis rank sum test. To examine how microbial compositions differed by plant parts and geographical ranges, permutational multivariate analysis of variance (PerMANOVAs, 9,999 permutations; Anderson, 2006) was conducted based on Bray-Curtis distance values with the “adonis2” function in “vegan.” We used ‘strata’ in adonis2 to control for site-to-site variation, as a random effect included in the PERMANOVA to restrict permutations solely within each country for range comparison. With Non-metric multidimensional scaling (NMDS) based on Bray–Curtis dissimilarity was also conducted within “vegan” and “ggplot2” (Wickham, 2016). In addition, Permutational analysis for multivariate homogeneity of dispersions was conducted for bacterial and fungal communities (PERMDISP, 9,999 permutations; Anderson, 2006). We also used sites as strata when as mentioned above. To compare the abundance of putative pathogens and mutualists between the two ranges, Welch’s t-test was conducted for the relative abundance of OTUs belonging to each FUNGuild. To find microbial genera biased to either the native or exotic range, we performed paired comparisons of genera that constituted more than 2% of the total relative abundance with Mann–Whitney U tests using the R package “exactRankTests” (Hothorn and Hornik, 2022).

## Results

### Fungal diversity in leaves, roots, and soil

The total numbers of fungal OTUs derived from leaves, roots and soil were 992, 1,244, and 2,415, respectively. The number of fungal OTUs detected only in the native range was 1,459 (36.5%), while 1,684 (42.1%) OTUs were specific to the exotic range, with 885 (21.4%) OTUs shared between the two regions. The leaf endophytic fungi were dominated by Ascomycota (90%; an average of the two ranges combined; Supplementary Figure S3A), whereas Basidiomycota accounted for a much smaller proportion (10%). The abundance of Ascomycota in the leaf was higher in the exotic range than in the native range (95 and 75%, respectively). Ascomycota was also dominant in the root and soil samples (57.4 and 38.3%, respectively), followed by Basidiomycetes (24 and 35%, respectively), and Mucoromycota (18 and 22%, respectively).

**Figure 2:**
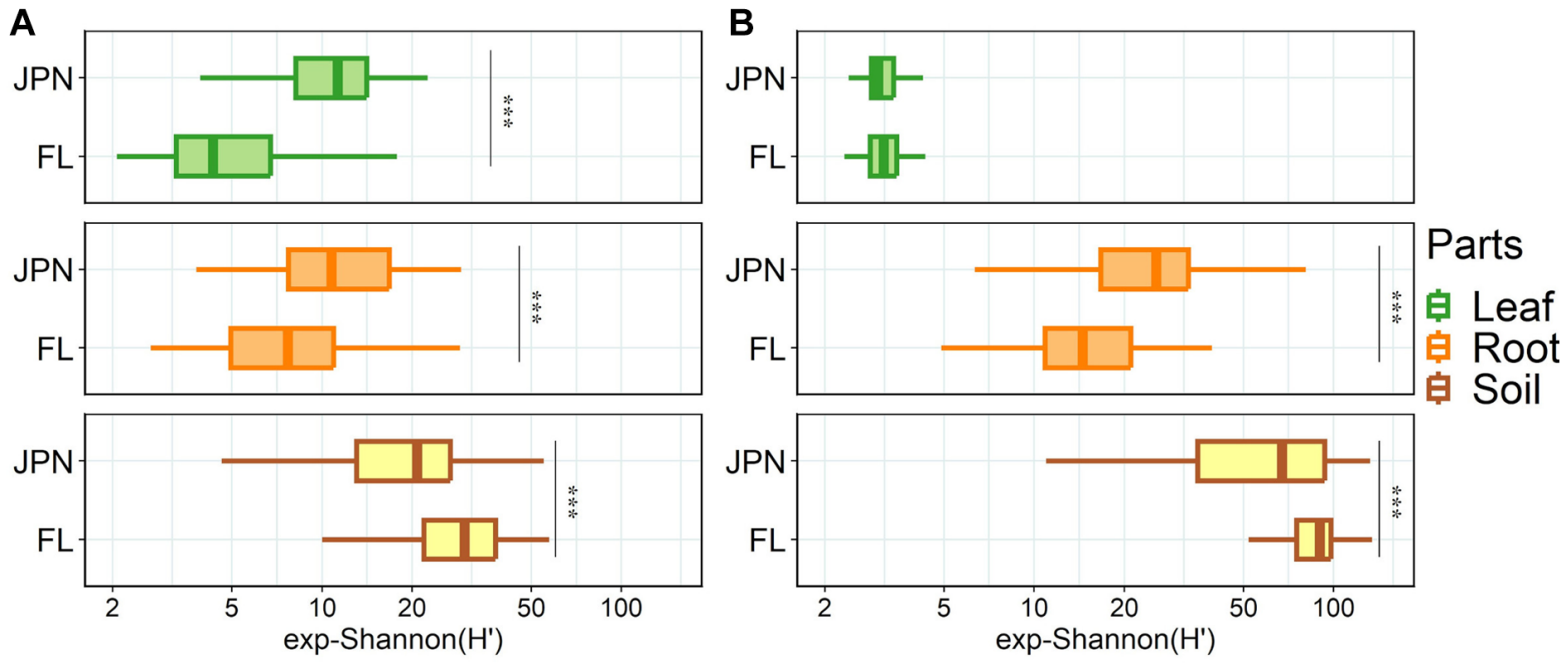
Shannon diversity of fungal **(A)** and bacterial **(B)** communities in Japan (JPN) and Florida (FL) sampled from leaves (top), roots (middle), and soil (bottom). Significant difference between locations as indicated by asterisks, determined by the non-parametric Kruskal-Wallis rank sum test (*p < 0.05, **p < 0.01, ***p < 0.001). See Supplementary Table S2 for each sample size.

Shannon diversity of fungal OTUs showed a significant interaction effect between 2 factors: plant parts and geographical range (ANOVA, *p* < 0.001, Supplementary Table S3). Additionally, there were significant differences among plant parts (ANOVA, *p* < 0.001), and small overall differences between the two geographical ranges (ANOVA, *p* = 0.685). Shannon diversity of fungi within leaves and roots were higher in the native range than in the exotic range, (the top two panels of Figure 2A), but it was higher in the exotic range than in the native range for soil (the bottom panel of Figure 2A).

### Bacterial diversity in leaves, roots, and soil

The total numbers of bacterial OTUs derived from leaf, root and soil samples were 490, 1,575 and 3,687, respectively. Among bacterial OTUs, 1,571 OTUs (32.6%) were detected only from the native range, whereas 1,300 OTUs (27.0%) were detected only from the exotic range, with 1,948 OTUs (40.4%) shared between the two regions. As to bacterial taxonomic composition, Proteobacteria was dominant in the leaf (Supplementary Figure S3B). *Burkholderia crenata*, an obligate symbiont taxon detected from *Ardisia crenata* previously (Carlier et al., 2016), was the most abundant Proteobacteria. In the root and soil, Proteobacteria and Actinobacteria dominated and accounted for more than half of the total OTUs.

Shannon diversity of bacterial OTUs had significant interaction effect between 2 factors: plant parts and geographical range (ANOVA, *p* < 0.001, Supplementary Table S3). Shannon diversity varied between the two geographical ranges (ANOVA, *p* = 0.002), and varied among the three plant parts (ANOVA, *p* < 0.001). Shannon diversity of the root-associated bacteria was higher in the native range than in the exotic range (middle panel, Figure 2B). In contrast, Shannon diversity in the -soil in the exotic range was higher than in the native range (bottom panel, Figure 2B).

### Fungal and bacterial community structures

**Figure 3:**
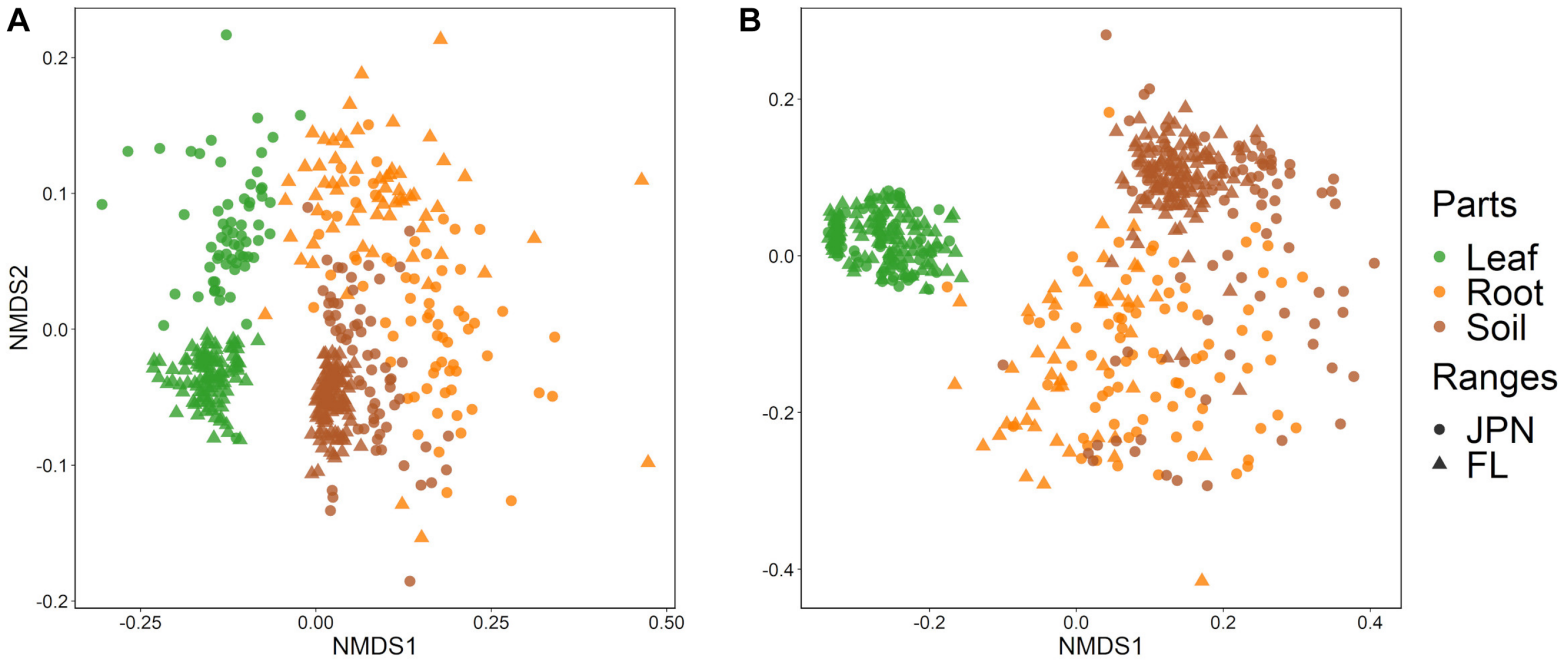
Nonmetric multidimensional scaling (NMDS) plots based on Bray–Curtis distances of **(A)** fungal and **(B)** bacterial communities sampled from leaves (green), roots (orange), and soil (brown). Each point represents individual of *A. crenata* sampled from Japan (〇) and Florida (△). Small numbers of obvious outliers were excluded from the figure (4 root samples of the fungal community, and 1 leaf sample and 4 root samples of the bacterial community). See Supplementary Table S4 for the results of PerMANOVA test on difference among parts and geographical ranges.

Non-metric multidimensional scaling (NMDS) plots showed strong differentiation of fungal and bacterial community structures among leaves, roots and soil, as well as between native and exotic regions (Figures 3A,B). The results of the PerMANOVA showed that plant parts and ranges had significant effects with strong interactions (Supplementary Table S4). Leaf endophytic fungi showed stronger differences between the native and exotic ranges than fungal communities in roots and soil (Figure 3A; Supplementary Table S5). Interactive effects between plant parts and geographical regions were also significant for bacterial communities (PerMANOVA, Supplementary Table S4). For bacteria, however, the regional difference was less pronounced in leaves compared to roots and soil (Supplementary Table S5), as visualized by the NMDS plot (Figure 3B). PERMADISP, which test whether the heterogeneity of OUT compositions differed between regions, showed significant differences (Supplementary Table S5). Greater community heterogeneity in the native range than in the exotic range was significant for fungal communities associated with leaves and roots (but not with soil), and for bacterial communities associated with rotos and soils (but not with leaves).

### Taxonomic comparisons between native and ranges

**Figure 4:**
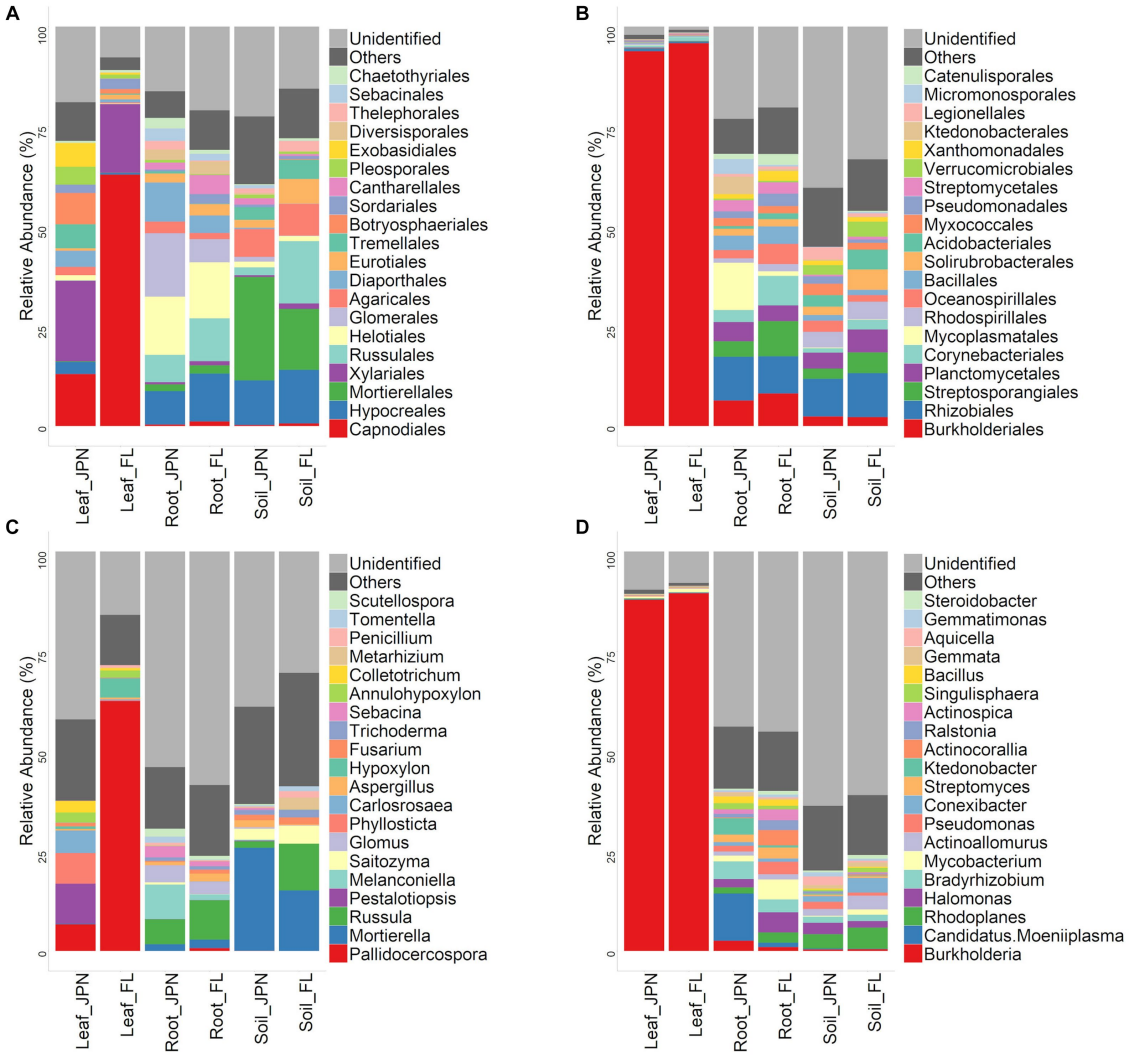
Taxonomic compositions of fungal OTUs (relative abundance) at the order **(A)** and genus **(C)** levels (left), and bacterial OTUs at the order **(B)** and genus **(D)** levels (right). The top 20 taxa are indicated, and all remaining taxa are consolidated into the ‘Others’ category.

Leaf endophytic fungi showed similar order-level diversity between the native and exotic ranges, with noticeable abundance of Capnodiales in the exotic range (Supplementary Figure S5A). At the genus level, *Pallidocercospora* dominated in the exotic range (62.5%, Figure 4C; Table 1), but *Pallidocercospora* was also common in the native range along with *Pestalotiopsis*, *Phyllosticta*, and *Carlosrosaea*. Order-level diversity of root-associated fungi was similar between the native and exotic ranges, with the same five orders dominant (Figure 4A). At the genus level, *Melanconiella* and *Russula*, and *Glomus* were the most common in the native range, whereas *Russula*, *Glomus*, and *Mortierella* were the most common in the exotic range (Figure 4C). Within the soil, Mortierellales was the most common in the native range, whereas its abundance was similar to those of Hypocreales (Figure 4A). In the exotic range, Russulales was also common. This reflects the abundance of *Russula*, *Saitozyma*, and *Metarhizium* in the exotic range, which was more pronounced than their abundance in the native range (Figure 4C). Order-level and genus-level fungal community compositions for each site were shown in Supplementary Figures S4A,C.

**Table 1:** List of fungal and bacterial genera exhibiting a prevalence of 2% or more in either native (Japan) or exotic range (Florida).

	Parts	Family	Genus	Guild	Proportion of reads (%)		*p* value
					Native	Exotic	
**Fungi**	**Leaf**	**Mycosphaerellaceae**	***Pallidocercospora***	**Plant Pathogen-Undefined Saprotroph**	**6.6**	**62.5**	**< 0.0001**
		**Sporocadaceae**	***Pestalotiopsis***	**Plant Pathogen**	**10.0**	**0.0**	**< 0.0001**
		**Botryosphaeriaceae**	***Phyllosticta***	**Plant Pathogen**	**7.7**	**0.1**	**< 0.0001**
		**Trimorphomycetaceae**	***Carlosrosaea***	**Unidentified**	**5.5**	**0.2**	**< 0.0001**
		**Hypoxylaceae**	***Hypoxylon***	**Unidentified**	**0.7**	**4.8**	**< 0.0001**
		**Hypoxylaceae**	***Annulohypoxylon***	**Unidentified**	**2.4**	**1.8**	**< 0.0001**
	**Root**	Russulaceae	*Russula*	Ectomycorrhizal	6.2	9.9	0.28
		**Melanconiellaceae**	***Melanconiella***	**Undefined Saprotroph**	**8.6**	**1.5**	**< 0.0001**
		**Glomeraceae**	***Glomus***	**Arbuscular Mycorrhizal**	**4.2**	**3.1**	**0.0004**
		Agaricomycetes	*Sebacina*	Unidentified	2.8	1.2	0.32
		Mortierellaceae	*Mortierella*	Endophyte-Litter Saprotroph-Soil Saprotroph-Undefined Saprotroph	1.7	2.1	0.29
	**Soil**	**Mortierellaceae**	***Mortierella***	**Endophyte-Litter Saprotroph-Soil Saprotroph-Undefined Saprotroph**	**25.9**	**15.1**	**< 0.0001**
		**Russulaceae**	***Russula***	**Ectomycorrhizal**	**1.6**	**11.7**	**< 0.0001**
		**Trimorphomycetaceae**	***Saitozyma***	**Unidentified**	**2.8**	**4.5**	**< 0.0001**
		**Clavicipitaceae**	***Metarhizium***	**Animal Pathogen**	**0.2**	**3.1**	**< 0.0001**
		Nectriaceae	*Fusarium*	Unidentified	1.4	1.8	0.05
**Bacteria**	**Leaf**	Burkholderiaceae	*Burkholderia*	-	93.0	94.5	0.37
		**Mycobacteriaceae**	***Mycobacterium***	**-**	**0.3**	**0.9**	**0.003**
		Hyphomicrobiaceae	*Rhodoplanes*	-	0.2	0.1	0.96
		Moraxellaceae	*Acinetobacter*	-	0.2	0.0	0.98
		Streptomycetaceae	*Streptomyces*	-	0.1	0.2	0.78
	**Root**	**Mycoplasmataceae**	**Candidatus.Moeniiplasma**	**-**	**11.5**	**1.3**	**< 0.0001**
		Burkholderiaceae	*Burkholderia*	-	5.5	4.9	0.97
		Bradyrhizobiaceae	*Bradyrhizobium*	-	4.3	3.3	0.02
		Halomonadaceae	*Halomonas*	-	2.2	5.0	0.91
		**Mycobacteriaceae**	***Mycobacterium***	**-**	**1.5**	**5.0**	**< 0.0001**
	**Soil**	**Hyphomicrobiaceae**	***Rhodoplanes***	**-**	**3.5**	**5.2**	**< 0.0001**
		**Conexibacteraceae**	***Conexibacter***	**-**	**1.4**	**3.9**	**< 0.0001**
		**Thermomonosporaceae**	***Actinoallomurus***	**-**	**1.6**	**3.4**	**< 0.0001**
		**Halomonadaceae**	***Halomonas***	**-**	**2.7**	**1.6**	**0.002**

Guild information was obtained through FUNGuild analysis. Note that OTUs assigned to more than one guild were treated as “Unidentified” in our analysis. Genera for which the Wilcoxon rank sum test between population ranges was significant are denoted in bold.

Order-level and genus-level composition of leaf endophytic bacteria were similar between native and exotic ranges, with a dominance of *Burkholderia crenata*, a symbiont in the leaf-edge nodules of *A. crenata* (Figures 4B,D; Table 1). At the order level, the composition of root-associated bacteria was dominated by Burkholderiales and Rhizobiales in both the native and exotic ranges. However, Mycoplasmatales were more common in the native range, while Streptosporangiales were more common in the exotic range (Figure 4B). At the genus level, *Candidatus.Moeniiplasma*, *Burkholderia*, and *Bradyrhizoblium* were most commonly found in the native range, while *Burkholderia*, *Mycobacterium*, and *Halomonas* dominated in the exotic range (Figure 4D). Within the soil, the order-level composition was similar between the native and exotic ranges, with Rhizobiales dominant in both. At the genus level, *Rhodoplanes* was the most common in both ranges, although its abundance was more pronounced in the exotic range. Order-level and genus-level fungal community compositions for each site were shown in Supplementary Figures S4B,D.

### Relative abundance of pathogen and mutualist guilds in native and exotic ranges

**Figure 5:**
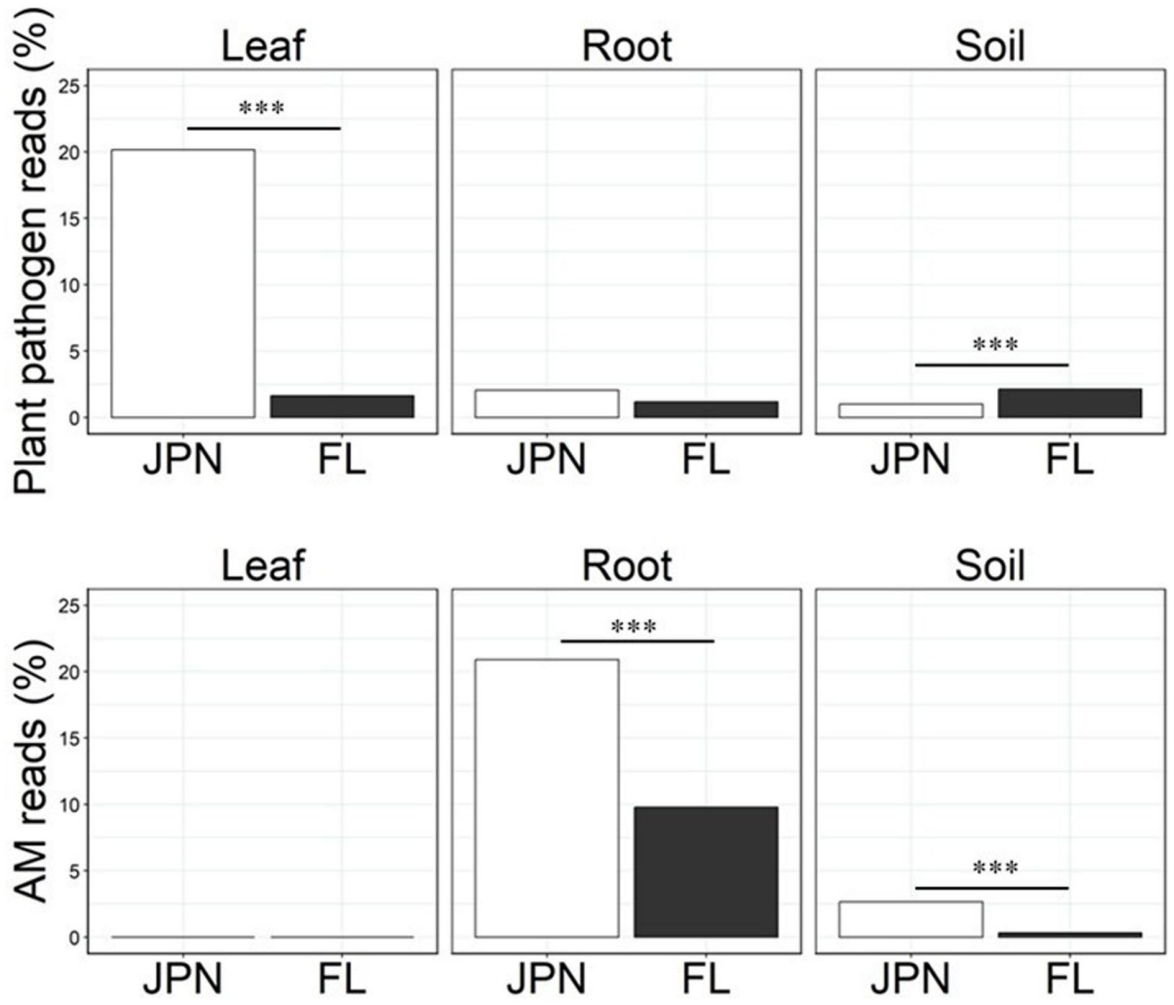
Relative abundance of fungal guilds, i.e., % of sequence reads that could be classified to plant pathogens (top) and arbuscular mycorrhizal (AM) fungi (bottom) within Japan (JPN) and Florida (FL) populations of *A. crenata* detected from leaf, root and soil samples. The guild classification was based on the FUNGuild at genus or family level. Asterisks indicates the results of Welch’s *t*-test (*p < 0.05, **p < 0.01, ***p < 0.001).

The relative abundance of plant pathogens differed significantly in leaf samples, with much higher relative abundance in the native range than in the exotic range (Figure 5, Welch’s *t*-test, *p* < 0.0001). However, there was no significant difference in the relative abundance of plant pathogens in roots (*p* = 0.349; Figure 5), and the relative abundance of pathogens in soil was similar but significantly higher in the exotic range than in the native range (*p* < 0.001; Figure 5). Among taxa that constituted more than 2% of the total relative abundance, *Pallidocercospora*, *Pestalotiopsis*, and *Phyllosticta* were assigned to plant pathogens (Table 1). *Pallidocercospora* was more common in the exotic range than in the native range, while *Pestalotiopsis* and *Phyllosticta* were unique in the native range (Figure 4). For *Phyllosticta*, 80% of the reads were confirmed to be *Phyllosticta ardisiicola* by the blastn top hit sequence in the NCBI database (Percent identity = 100%; accession number; NR136952.1). *P. ardisiicola* is reported to be a pathogen discovered from *A. crenata*, causing leaf spots in *A. crenata* (Motohashi et al., 2008). A literature review was conducted on microbial taxa with high abundance (> 2% of the total), but no candidate pathogenic microbes were found.

The relative abundance of AM fungi in roots and soil was significantly higher in the native range than in the exotic range by 114 and 706%, respectively (Welch’s t-test, *p* < 0.0001 each, Figure 5, bottom). *Glomus and Russula* were identified as mutualistic microbes among taxa that constituted more than 2% of the total relative abundance (Table 1). *Glomus* is a major group of arbuscular mycorrhizal fungi (Morton and Benny, 1990), and *Russula* is considered to be ectomycorrhizal fungi (Smith and Read, 2010). *Glomus* was more common in the native range than in the exotic range, while the genus *Russula* was less common in the native range than in the exotic range. Our literature review of microbial taxa with high abundance (> 2% of the total) found no clear candidates that may act as mutualists in leaves, except for *Carlosrosaea*. This genus was more common in the native range than in the exotic range, and it has been reported as a potentially mutualistic endophyte that improves seedling growth of Bromeliaceae (Marques et al., 2021).

## Discussion

To explore potential differences in microbial communities associated with the invasion success of *A. crenata*, we compared fungal and bacterial community structures within the leaf, root, and soil between native and exotic ranges. Two trends stood out. Firstly, the microbial diversity inside leaves and roots was lower in the exotic range than that in the native range, despite higher microbial diversity in the soil in the former. Secondly, leaves harbored a higher number of plant pathogenic fungal genera in the native range than in the exotic range, which is corroborative of the enemy release hypothesis. These findings underscore the importance of microbial diversity including potential pathogens in keeping check on the host plant population in the native range.

### Microbial composition in leaf, root, and soil in the native and exotic ranges

Previous studies that examined either fungi or bacteria associated with invasive plants reported differences in microbial community between plant parts or population ranges (Gundale et al., 2016; Pickett et al., 2022). Our study that examined both fungi and bacteria is more comprehensive in revealing differences among leaves, roots and soils, as well as between geographical ranges (Figures 3A,B). Lu-Irving et al. (2019) compared bacterial communities of invasive grass between its native and exotic ranges, and reported lower bacterial diversity in the leaf endosphere, root endosphere, and root surface in the exotic range than in the native range, while some research indicates the opposite, or shows no clear trend (Pickett et al., 2022; Pan et al., 2023). These inconsistent results might be dependent on the invasive plant species under consideration.

The composition and diversity of leaf endophytic bacteria were similar between the native and exotic ranges, with strong dominance of *Burkholderia crenata*, which is known to be an obligate symbiont that is vertically transmitted from mother plants (Ku and Hu, 2014). Perhaps, this bacterium is maintained throughout the process of cultivation and subsequent naturalization of *A. crenata* in the US. In contrast, the composition of leaf endophytic fungi showed wide differences by geographical ranges compared to leaf endophytic bacteria and fungi in roots and soil (Figures 3A,B; Supplementary Table S5), suggesting that *A. crenata* interacts with distinct fungal communities in its exotic range as opposed to its native range. We found a lower diversity of leaf endophytic fungi in the exotic range than in the native range, possibly tied to the dominance of a latent plant pathogen, *Pallidocercospolla*, in the exotic range (Figure 4C). Previous studies have reported cases of accumulation of specific plant pathogens in invasive plants in their exotic ranges (Stricker et al., 2016; Anthony et al., 2017). *Pallidocercospolla*, which is obviously non-lethal, may have accumulated in the leaf of *A. crenata* as its population density increased within the exotic range.

Differences in the root fungal and bacterial taxonomic composition were observed between the native and exotic ranges, although these variations were less pronounced than those in the leaves or soil (Supplementary Table S5). This could be attributed to a higher microbial diversity in roots (i.e., wider scatter Figures 3A,B), compared to that in leaf and soil samples. There are two potential factors for explaining such pattern: (1) colonization of microbes into roots could vary among individual plants; (2) microbial colonization of roots varies greatly among fine roots within individuals (Rüger et al., 2021). We cannot distinguish these two possibilities as we sampled only a few fine root samples per plant. Overall, the diversity of root-associated microbes in the exotic range was lower than in the native range, despite more diverse soil microbial pool than in the native range (Figures 2A,B). Considering this, one possibility is that root-associated microbes are selectively accumulated by *A. crenata* rather than passively recruited from the locally available species pool in the soil. This could be due to the host plants imposing selection or because fewer microbes in exotic ranges can overcome host resistance.

Soil microbial communities differed significantly between native and exotic ranges for both bacteria and fungi, similar to the findings of a previous study comparing geographical ranges of invasive grasses (Lu-Irving et al., 2019). These differences could merely reflect biogeographical differences between Japan and Florida, and/or the possible influence of invasive plants on soil microbial communities (Trognitz et al., 2016; Rodríguez-Caballero et al., 2020). The local monodominance of invasive exotic plants may influence microbial communities along with decreasing species richness of aboveground plant communities (Anthony et al., 2017; Zhang et al., 2020). Contrary to our hypotheses, both fungal and bacterial diversity of soil were higher in the exotic range than in the native range. According to Ramirez et al. (2019), invasive species can produce more root exudates when they are in environments without their natural predators. This increased production of root exudates might contribute to a rise in the diversity of microbes in the soil. These possibilities are worth testing to improve mechanistic understanding of the geographical variances of microbial composition and diversity.

### Support for enemy release hypothesis

Many studies examining the enemy release hypothesis have focused on belowground pathogens or aboveground herbivores (Adams et al., 2009; Chiuffo et al., 2015; Aldorfová et al., 2020; Huang et al., 2020). Whereas less attention has been given by the research community to leaf endophytic microbes in the context of the enemy release hypothesis, the results of our study suggest the potential importance of leaf endophytic fungi. Indeed, we found a sign of enemy release only in leaf endophytic fungi, but not in bacteria or root-associated fungi (Figure 5; Table 1). Notably, the pathogenic fungi observed in the leaf endosphere of the native population were diverse, including *Pestalotiopsis* with a wide host range (Maharachchikumbura et al., 2014), and *Phyllosticta ardisiicola* known to cause specific diseases in *A. crenata* (Motohashi et al., 2008). These deleterious fungi were found only in the native range (Japan) but absent in the exotic range (Florida). Hence, they deserve further study as potential candidates that may limit the local density of *A. crenata* in Japan. On the other hand, *Pallidocercospora*, a known pathogenic genus, was more abundant in leaves sampled in the exotic range (Crous et al., 2013). However, *Pallidocercospora* is often detected in healthy leaves and hence it has been suggested to be a latent pathogen (Napitupulu et al., 2021). We suspect that it is only weakly deleterious and the high local density of *A. crenata* in the exotic range may promote its abundance. To explore this possibility, we are currently conducting another study to examine the effect of local population density of *A. crenata* within the exotic range of Florida. Overall, we detected patterns in support of the enemy release hypothesis with leaf endophytic fungi than with leaf endophytic bacteria, or fungi and bacteria associated found in roots or soils.

### Support for enhanced mutualism hypothesis

We did not observe microbial patterns that are supportive of the enhanced mutualism hypothesis either for fungi, as the relative abundance of arbuscular mycorrhizal (AM) fungi within roots was higher in the native range than in the exotic range (Figure 5). Several other studies on the acquisition of new AM fungi report higher AM fungal colonization rates and greater AM fungi diversity in the exotic range compared to the native range (Yang et al., 2013; Soti et al., 2014; Sheng et al., 2022). *A. crenata* is reported to be capable of acquiring genotypes of AM fungi that enhance growth in Florida (Bray et al., 2003). Even though many AM fungi are considered generalist, their effectiveness depends on the combination of host and fungal species and genotypes. Our study found greater abundance of AM fungi within the roots of plants from the native range than those from the exotic range (Figure 5; Table 1), but it is not clear whether they are necessarily effective mutualists. Although ectomycorrhizal fungi (EcM) were more common in the exotic range soils (largely attributable to the genus *Russula*), the relative abundance of OTUs assigned to EcM in the roots was similar between native and exotic ranges (Table 1). *A. crenata* is not known to form symbiotic relationships with EcM fungi: there are no reports suggesting an interaction between *A. crenata* and EcM, and we do not detect EcM hyphae inside the roots under microscopes (data not shown). The high relative abundance of EcM in exotic range soils is likely to reflect differences in the overstory vegetation. Whereas ectomycorrhizal pines and oaks were dominant in Florida sites, AM fungi dependent conifers (cedars and cypress) were mixed with oaks in Japan (Supplementary Table S1). For bacterial communities, we did not find any notable differences in abundance of potentially beneficial bacteria between native and exotic ranges, with ubiquitously high abundance of Burkholderia in leaves.

## Conclusion

Whereas many recent studies addressed positive and negative feedbacks between plants and soil microbial communities, our results suggest that it is essential to simultaneously examine leaf-associated microbial communities. A vast diversity of microbes were found to interact with *A. crenata* in both native and exotic ranges, including mutualistic, commensalistic, to pathogenic fungi and bacteria. While functional guilds were estimated from the database, a given microbe may act differently depending on environmental and host conditions. Furthermore, these microbes interact with each other in addition to their direct interaction with their host plant. We did not evaluate the interactions between hosts and microbes, but narrowed down candidates that may cause ecologically significant interactions with *A. crenata* in its native and exotic ranges. Specifically, the results suggest a potential importance of leaf pathogenic fungi in explaining the local density of *A. crenata* in Japan vs. Florida. Manipulative experimental study that employs density manipulation and inoculation tests with these putative pathogens within the native range of *A. crenata* will prove whether these are the key density-dependent agents, the lack of which explains the invasive population growth in the exotic range.

## Data availability statement

The datasets presented in this study can be found in online repositories. The names of the repository/repositories and accession number(s) can be found at: https://ddbj.nig.ac.jp/public/ddbj_database/dra/fastq/, DRA017027.

## Author contributions

NN: Investigation, Writing - original draft, Conceptualization, Validation. HT: Writing - review & editing, Conceptualization, Validation. KK: Writing - review & editing, Conceptualization, Validation.
