# Supplementary material for: Leaf, root, and soil microbiomes of an invasive plant, Ardisia crenata, differ between its native and exotic ranges
Source: Front Microbiol. 2023 Nov 22;14:1302167. doi: 10.3389/fmicb.2023.1302167 (PMC10703299; doi:10.3389/fmicb.2023.1302167)
Supplement: Supplementary file 1 [file Data_Sheet_1.docx]

Supplementary Material

Leaf, root and soil microbiomes of an invasive plant, *Ardisia*

*crenata*, differ between its native and exotic ranges

Naoto Nakamura ^1^ *, Hirokazu Toju ^2^, Kaoru Kitajima ^1^

^1^ Faculty of Agriculture, Kyoto University, Kyoto Japan

^2^ Center for Ecological Research, Kyoto University, Otsu, Japan

*** Correspondence:**Naoto Nakamura E-mail address: [nakamura.naoto.35c@st.kyoto-u.ac.jp](mailto:nakamura.naoto.35c@st.kyoto-u.ac.jp)

SUPPLEMENTARY FIGURE


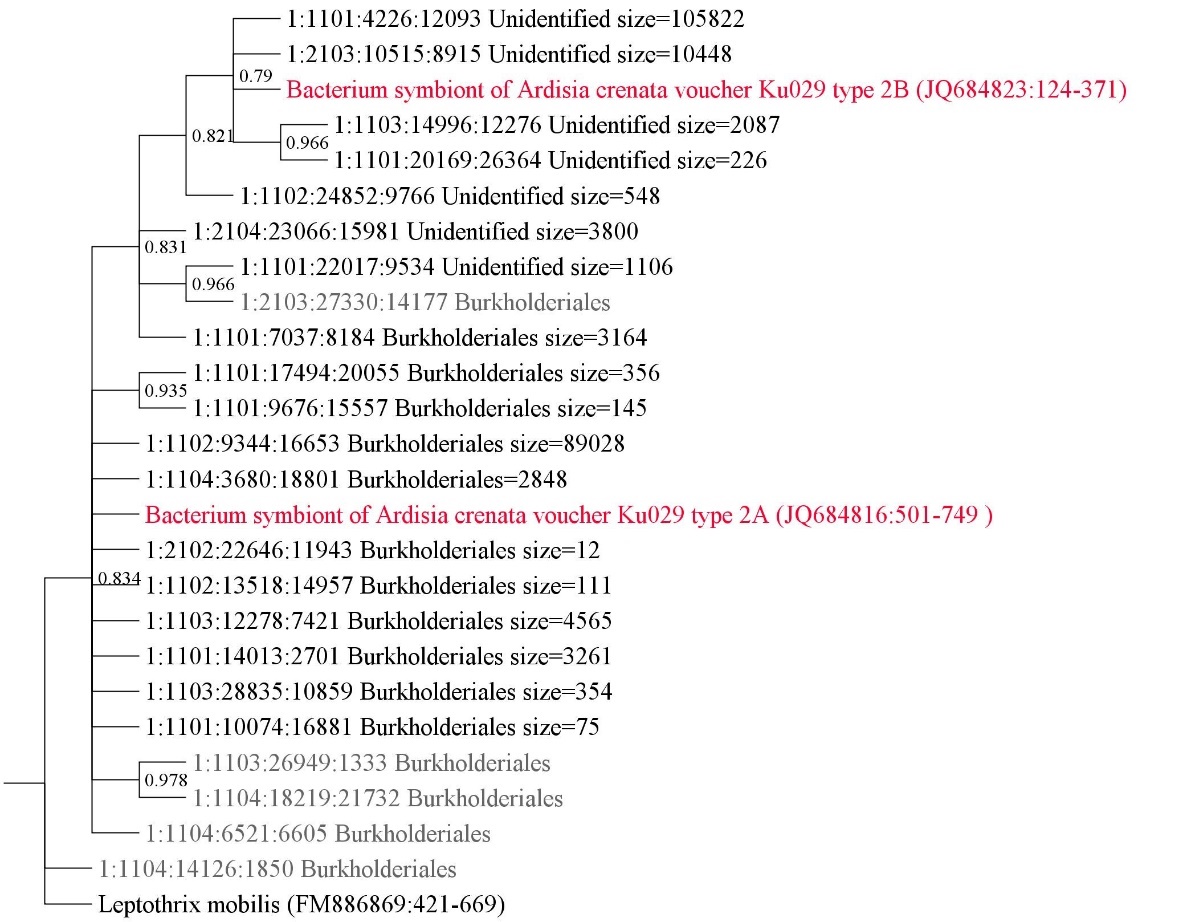


Supplementary Figure S1

Bootstrap consensus phylogenetic tree constructed based on the analysis of the 16S rDNA sequences of OTUs presumed to be symbionts of *A. crenata*. For the phylogenetic analysis, OTUs that matched either 2 types of the reference sequences of *Burkholderia crenata* (JQ684816 and JQ684823) in the 806b-515f region with a similarity of 95% or higher were included. The search was conducted with VSEARCH (Rognes et al., 2016), and as a result, 18 OTUs out of a total of 8295 OTUs were matched. In addition to the 18 OTUs, 2 reference sequences (red) and 5 sequences of Buekholderiales OTUs randomly chosen from our dataset (grey) were included for estimating phylogeny. As an outgroup, 806b-515f region of *Leptothrix mobilis*, belonging to the Burkholderiales were used (FM886869). The software "muscle" (Edgar et al., 2004) and "Seaview" (N.Galtier, 1996) were employed for DNA sequence alignment, and the Neighbor-joining method with MEGA version 11 (Tamura et al., 2021) was used to estimate and visualize phylogeny. Numbers adjacent to branches represent bootstrap support levels based on 1,000 resampled datasets. Branches with lower support than 75 % were collapsed. The Order-level taxonomic identification is indicated at the tips, accompanied by the read count obtained from Illumina MiSeq sequencing. Four unidentified OTUs with read counts of 105822, 10448, 2087, and 266 were grouped with the branch containing reference sequences of referenced sequence, so we labeled　them as “*Burkholderia*. *sp*” in our dataset. Additionally, three other unidentified OTUs with read counts of 548, 3800, and 1106 were labeled as “Burkholderiales”.


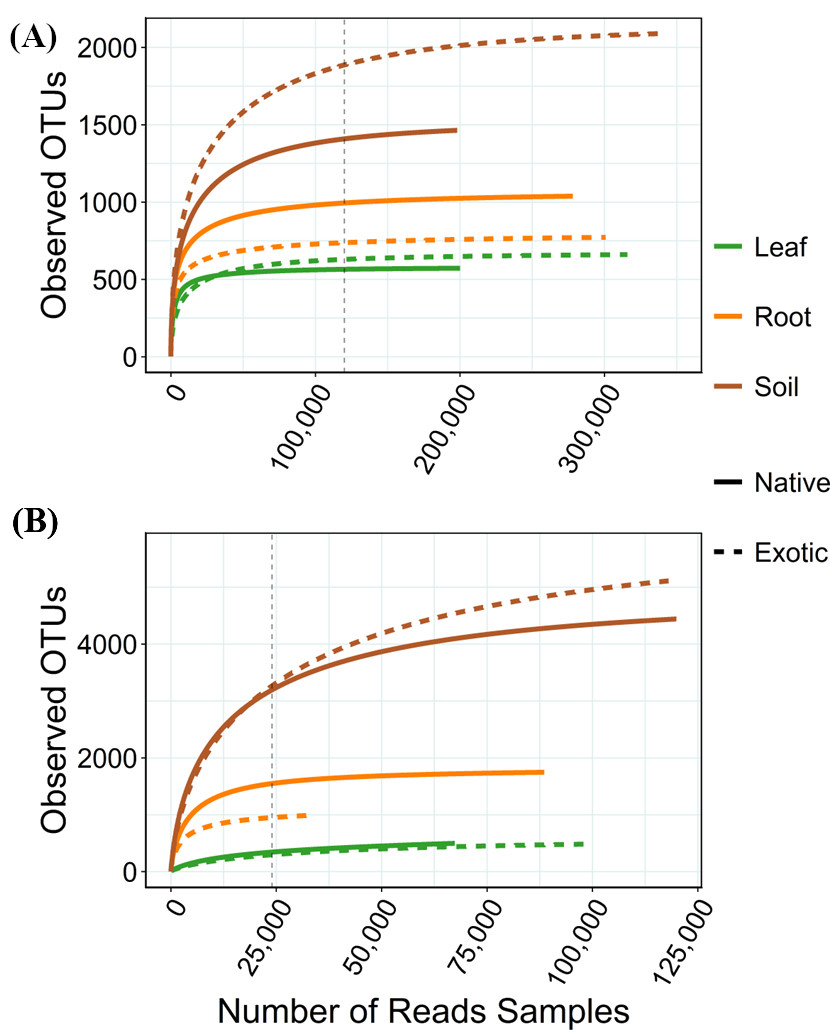
Supplementary Figure S2

Species accumulation (rarefaction) curves of detected OTUs from ITS (A, fungi) and 16S (B, bacteria) from samples pooled by plant part and population range. Colors represent leaf (green), root (orange) and soil (brown) samples. Solid and dashed lines represent samples from native and exotic populations, respectively. Vertical doted lines indicate the sample sizes rarefied for the analysis reported.


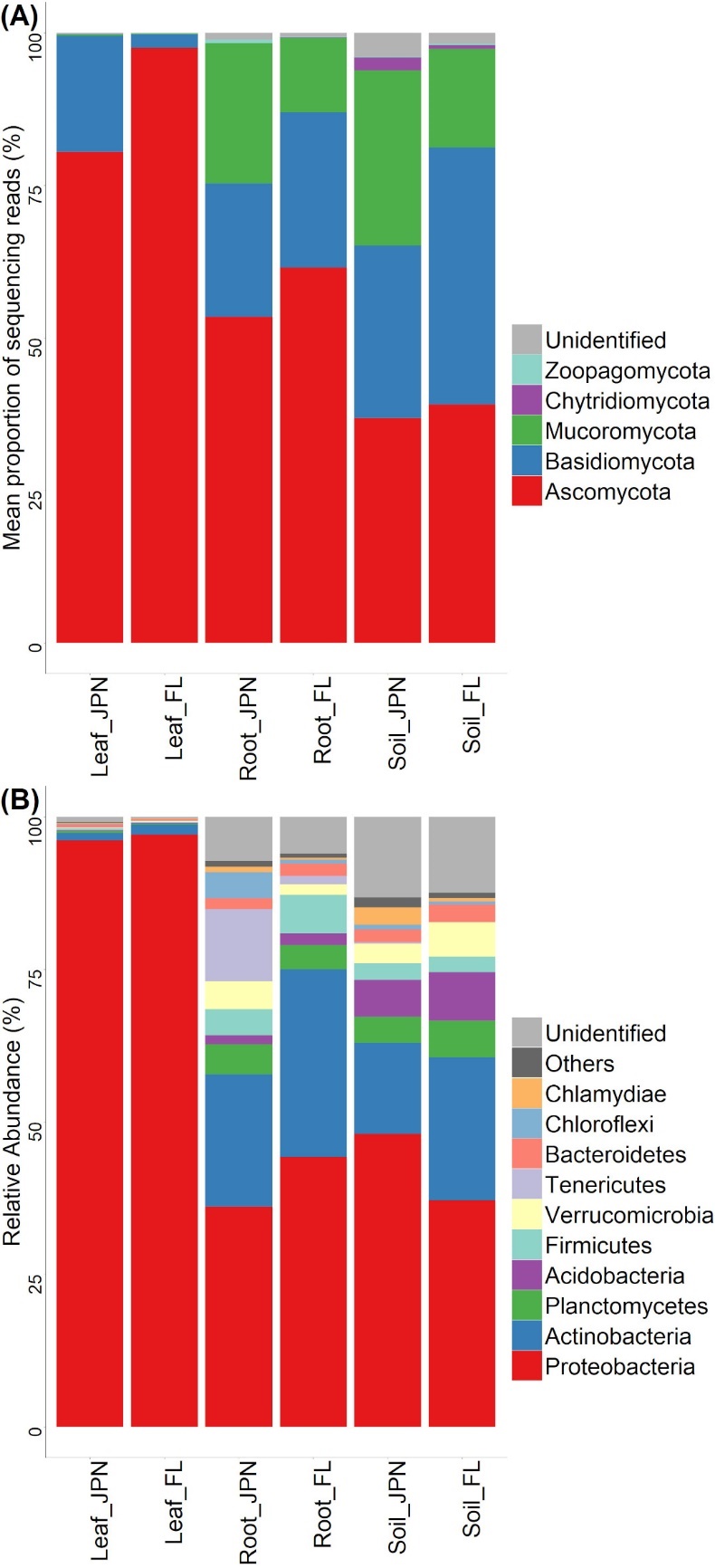


Supplementary Figure S3

The phylum-level taxonomic omposition of fungal (A) and bacterial (B) OTUs. The top 5 taxa for bacteria and the top 10 taxa for fungi were displayed All remaining species are consolidated into the 'Others' category. The vertical axis indicates the relative abundance of each taxon.


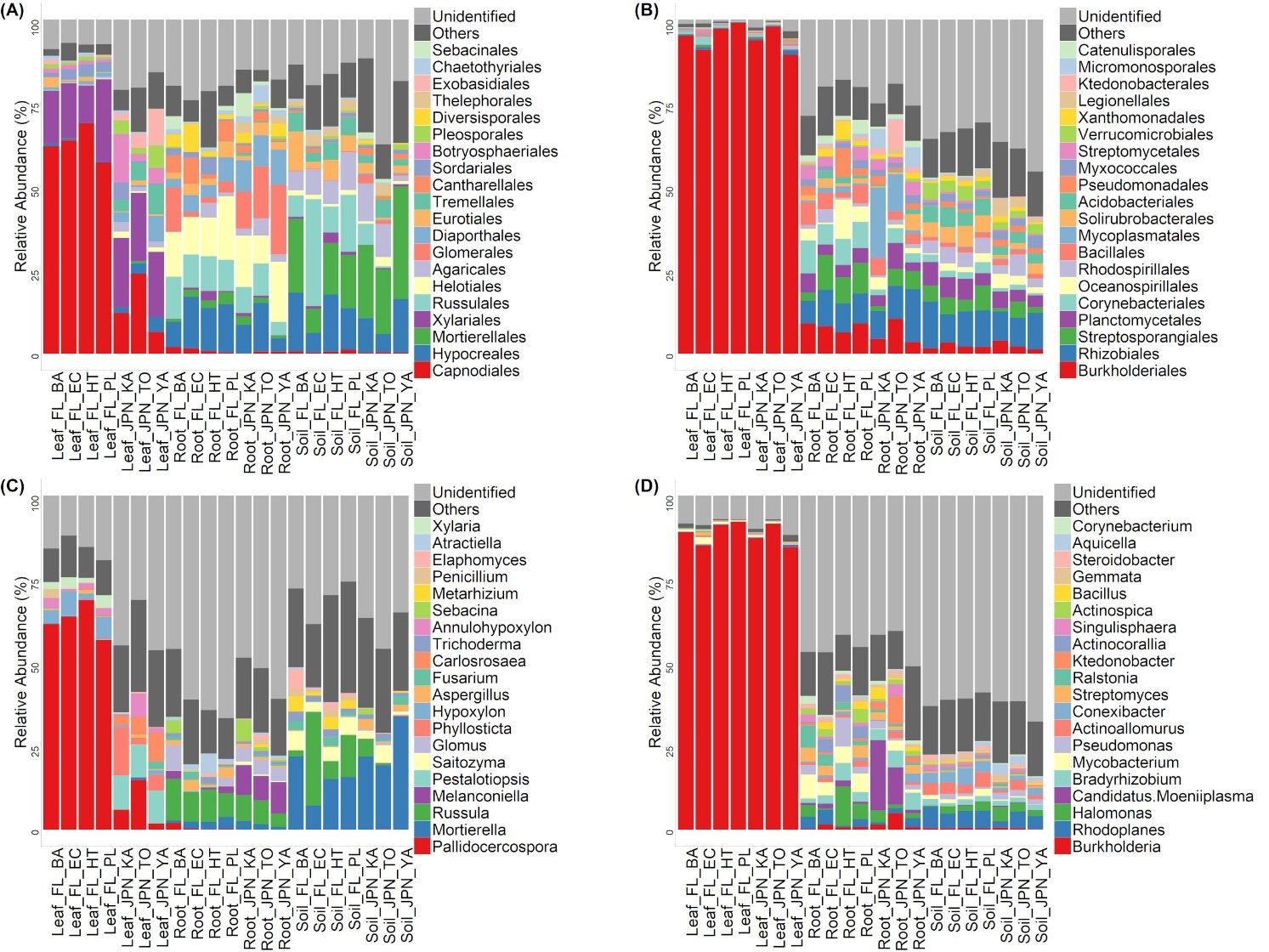


Supplementary Figure S4

Taxonomic compositions of fungal OTUs (relative abundance) at the order (A) and genus (C) levels (left), and bacterial OTUs at the order (B) and genus (D) levels (right) for each sampling site. The top 20 taxa are indicated, and all remaining taxa are consolidated into the 'Others' category. See Supplementary Table S1 for geographical coordinates and vegetation characteristics of sampling sites.

Reference

Edgar, R. C., Drive, R. M., and Valley, M. (2004). MUSCLE : multiple sequence alignment with high accuracy and high throughput. 32, 1792–1797. doi: 10.1093/nar/gkh340.

N.Galtier (1996). SEA VIEW and PHYLO_ WIN: two graphic tools for sequence alignment and molecular phylogeny. 12, 543–548.

Rognes, T., Flouri, T., Nichols, B., Quince, C., and Mahé, F. (2016). VSEARCH: a versatile open source tool for metagenomics. *PeerJ* 4, e2584. doi: 10.7717/peerj.2584.

Tamura, K., Stecher, G., and Kumar, S. (2021). MEGA11: Molecular Evolutionary Genetics Analysis Version 11. *Mol. Biol. Evol.* 38, 3022–3027. doi: 10.1093/molbev/msab120.
